# Cross-material physics-informed machine learning framework for optimizing nanofiller loading in epoxy nanocomposites for high-voltage insulation

**DOI:** 10.1038/s41598-026-61831-w

**Published:** 2026-07-17

**Authors:** Mahmoud Ezzat, M. Ramadan, Mousa. A. Abd-Allah, S. M. A. El-Gamal, Abdelrahman Said

**Affiliations:** 1 Department of Electrical Engineering, Faculty of Engineering at Shoubra, Benha University, Cairo, 11672 Egypt; 2 Chemistry Department, Faculty of Science, Ain Shams University, Cairo, 11566 Egypt; 3 Faculty of Computer Science, Benha National University (BNU), Al Obour, Egypt

**Keywords:** Epoxy nanocomposites, Dielectric breakdown strength, Machine learning, Layered double hydroxide, Recycled alumina nanofillers, Engineering, Materials science, Nanoscience and technology

## Abstract

Epoxy-based nanocomposites are promising solid insulation materials for high-voltage applications because of their high dielectric strength, mechanical robustness, and processability. However, identifying the optimal nanofiller loading that maximizes dielectric breakdown strength (BDS) remains challenging because conventional trial-and-error approaches are costly, time-consuming, and difficult to generalize across material systems. This study proposes a cross-material physics-informed machine learning framework integrating four structurally distinct nanofillers: Zn/Al-LDH, Mg/Al-LDH, γ-Al_2_O_3_, and α-Al_2_O_3_, where the alumina nanoparticles were synthesized from recycled aluminum beverage cans as a sustainable material source. For each system, 15 breakdown measurements per concentration were statistically validated using Weibull analysis. Among all systems, α-Al_2_O_3_ exhibited the highest BDS of 46.8 kV/mm at 5 wt%, corresponding to approximately 56% improvement over neat epoxy. Dataset augmentation was performed using PCHIP interpolation combined with Gaussian noise injection and validated through Leave-One-Out Reconstruction analysis, which showed interpolation errors below 7.76% for internal concentration points. Composite relative permittivity at intermediate concentrations was estimated using the Maxwell–Garnett model and incorporated as a physically constrained input feature. Five regression algorithms were trained and benchmarked on material-specific datasets, achieving R^2^ values up to 0.959 with experimental validation errors below 5.2%. A cross-material model was further developed by replacing categorical material identity with intrinsic filler permittivity as a physics-based descriptor, enabling a transferable across multiple investigated nanofiller systems using a common descriptor. The cross-material model achieved R^2^ = 0.919 with prediction errors below 6%. The proposed framework provides a scalable and experimentally validated route for optimizing epoxy insulation systems for GIS/GIL spacer applications.

## Introduction

The development of advanced insulating materials remains a major priority in electrical engineering, particularly in high-voltage applications. Reliable insulation systems must exhibit high dielectric strength, mechanical robustness, and high environmental resistance to ensure continuous and safe operation^1,2^. Traditionally, inorganic materials such as porcelain and glass have been widely used because of their chemical stability and their ability to withstand electrical arcing and surface degradation^3^. However, in recent decades, polymer-based materials, especially epoxy resins and silicone rubbers, have increasingly replaced conventional insulators because of their lighter weight, easier processing, and tunable electrical and thermal properties^4–7^.

Incorporating nanomaterials into epoxy resins significantly affects their electrical and thermal performance. At the dielectric scale, the introduction of nanoparticles generates extensive interfacial regions that alter the charge transport dynamics and polarization processes, resulting in lower permittivity and dielectric losses at moderate filler concentrations compared with conventional micro-composites^8,9^.

In gas-insulated systems such as GIS and GIL, epoxy spacers play a critical role in ensuring insulation reliability. Their performance depends not only on the breakdown strength, which defines the failure limit, but also on the relative permittivity, which governs the electric field distribution within the system^10^.

A mismatch in permittivity between the spacer and surrounding gas can cause local field enhancement at the gas–solid interface, leading to partial discharge and surface flashover. Therefore, optimizing the breakdown strength alone is insufficient; it must be achieved along with controlled permittivity to ensure uniform field distribution and reliable operation^11^.

Extensive research on epoxy nanocomposites has revealed that oxide nanofillers strongly modulate dielectric and thermal behavior depending on their concentration. In silica-filled systems, low loading (< 5 wt%) slightly increased permittivity and conductivity, whereas intermediate contents (5–15 wt%) promoted interfacial immobilization that restricted dipolar motion and reduced dielectric loss; beyond ~ 20 wt%, percolation phenomena caused a sharp rise in conductivity and permittivity^9^.

Comparable trends were observed in the epoxy/Al_2_O_3_ nanocomposites, where the dielectric loss reached its minimum around 5 wt% and both permittivity and breakdown strength exhibited non-linear variation with filler content^12–14^.

Producing composite materials with targeted dielectric and mechanical properties often requires the preparation of many samples, which substantially increases experimental cost and time. To overcome these limitations, Pilania et al. employed advanced predictive algorithms combined with quasi-experimental datasets to model the material behavior^15^. This strategy replaces the inefficiencies of conventional trial-and-error experimentation with data-driven mathematical models that are capable of accurately forecasting material performance.

Machine learning (ML) has rapidly evolved into a transformative approach in materials science, offering powerful tools for analyzing complex relationships between structure, processing, and properties. By leveraging relatively small and well-curated datasets, ML algorithms can construct predictive correlations that accelerate materials design and discovery. By integrating generative models and inverse-design techniques, properties can now be estimated and optimized with significantly fewer experimental iterations^16^.

Various regression-based algorithms have been utilized to predict material characteristics, including Support Vector Machines (SVM), K-Nearest Neighbors (KNN), Gradient Boosting Machines (GBM), Random Forests (RF), Decision Trees (DT), Extra Trees (ET), and Extreme Gradient Boosting (XGBoost)^17^. Siddique et al. examined the influence of different doping concentrations on the dielectric behavior of epoxy-based nanocomposite^18^.

In this study, a generalized, physics-informed machine learning framework is proposed to predict the optimal nanofiller loading that maximizes the dielectric breakdown strength of epoxy-based nanocomposites. Despite extensive research on epoxy nanocomposites, most existing studies rely on discrete experimental measurements and trial-and-error approaches to identify optimal filler concentrations. Such approaches are time-consuming and do not provide a continuous or generalizable understanding of the relationship between filler loading and dielectric performance^19,20^.

Moreover, current studies are typically limited to specific material systems, with no unified framework capable of predicting the dielectric behavior across different classes of nanofillers using physically meaningful descriptors. In particular, the role of intrinsic filler properties; such as permittivity has rarely been incorporated into predictive modelling.

To address these limitations, the present work investigates two major classes of nanofillers: layered double hydroxides (LDHs) and alumina-based nanoparticles, representing distinct material families with different dielectric characteristics. Experimental measurements were used as a validated baseline, upon which data processing and machine learning models were developed.

Material-specific models were first constructed to capture the behavior of individual systems, followed by the development of a cross-material model based on intrinsic filler permittivity. This enables the transition from material-dependent prediction to a unified descriptor-based framework capable of learning transferable relationships across the investigated nanofiller systems.

The proposed approach provides a scalable and data-driven methodology for predicting optimal filler concentrations with high accuracy, significantly reducing the experimental effort and offering practical guidance for the design of advanced epoxy insulation materials for high-voltage applications.

## Materials

Four types of nanomaterials were prepared and employed as fillers: Zn/Al layered double hydroxide (Zn/Al–CO_3_ LDH), Mg/Al layered double hydroxide (Mg/Al–CO_3_ LDH), and α/γ-alumina (Al_2_O_3_) obtained from recycled aluminum cans.

Layered double hydroxides were synthesized using the co-precipitation method. Equimolar solutions of metal salts [Zn(NO_3_)_2_·6 H_2_O or Mg(NO_3_)_2_·6 H_2_O, 0.5 M] and AlCl_3_ (0.5 M) were prepared in a 2:1 molar ratio and stirred magnetically for 15 min to ensure homogeneity. A 2 M Na_2_CO_3_ solution was added dropwise until a white gel-like precipitate was formed. The pH was maintained at 7–8 for the Zn/Al-LDH and 9–10 for the Mg/Al-LDH. The obtained precipitates were filtered, washed repeatedly with distilled water, dried overnight at 80 °C, and then ground and sieved to yield fine LDH powder^11,21^.

Alumina nanoparticles were prepared from recycled aluminum beverage cans. The cans were first cleaned to remove paint and impurities, then dissolved into hydrochloric acid to form an aluminum chloride solution. Precipitation of aluminum hydroxide was induced using sodium carbonate, followed by filtration and washing. The resulting hydroxide was calcined at 600 °C to obtain γ-Al_2_O_3_ and at 1200 °C to obtain α-Al_2_O_3_ nanoparticles^22,23^.

Surface functionalization was employed to improve the compatibility and dispersibility of the nanoparticles within the epoxy matrix. Chemical modification using silane coupling agents, such as 3-aminopropyltriethoxysilane (APTES), was adopted because of its ability to form stable bonds with both the nanoparticle surface and polymer chains, thereby reducing hydrophilicity and promoting uniform dispersion^3,21^.

The epoxy-based nanocomposites were prepared by incorporating functionalized nanoparticles at different loadings (1, 3, 5, and 7 wt%). The dried nanoparticles were first ultrasonicated in ethanol to break the agglomerates and achieve uniform dispersion, then gradually introduced into the epoxy resin under mechanical stirring and further ultrasonication to enhance homogeneity. The residual solvent was removed by heating the mixture at 80 °C until a constant weight was achieved. The nanoparticle–epoxy mixture was subsequently blended with a curing agent (3:1 weight ratio), degassed to eliminate trapped air bubbles, and cast into pre-prepared molds. Curing was performed under ambient conditions for 24 h, followed by post-curing by heating the samples for 3 h at 120 °C. The specimens obtained were then used for dielectric and AC breakdown measurements^9,21,23^.

## Methodology

In this study, a systematic framework was established to predict the optimal nanofiller concentration for enhancing the breakdown strength of epoxy-based nanocomposites using four different types of nanofillers. The methodology combines controlled experimental measurements, statistical data augmentation, and machine learning (ML) modelling in an integrated workflow.

The epoxy nanocomposites were prepared following a stepwise loading strategy, in which the nanofiller was incorporated incrementally rather than preparing all concentrations simultaneously. At each step, a defined filler loading was introduced into the epoxy matrix, cured, and tested. This incremental approach allowed continuous tracking of the breakdown strength trend; therefore, the loading was increased as long as improvement occurred, and the process was halted once a decline was observed. Thus, the optimum loading range was efficiently localized without unnecessary sample preparation^21,23^.

The AC breakdown strength of the epoxy nanocomposites was evaluated using a standardized oil-immersion test setup in accordance with ASTM-D149. Disk-shaped specimens (1 mm thick) were placed between the spherical electrodes and fully immersed in insulating oil to suppress surface flashover, ensuring that the failure corresponded to intrinsic dielectric breakdown. A uniform voltage ramp of 500 V/s was applied until breakdown occurred, and multiple specimens were tested at each loading level to ensure statistical reliability of the results^21,23^.

Although the breakdown strength measurements were conducted in accordance with ASTM-D149, which typically reports five breakdown tests per loading, 15 independent measurements were conducted for each concentration in this study. This strategy was adopted to capture the inherent stochastic nature of dielectric breakdown, reduce experimental uncertainty, and overcome the limitations of earlier reports that lacked sufficient statistical representation. Furthermore, by generating a richer experimental dataset, subsequent data augmentation and machine learning analyses could be performed on a more reliable basis, ensuring that the synthetic data derived from interpolation faithfully reflected the underlying variability of the material.

A data augmentation strategy was applied to overcome the limitations of having measurements at only four concentrations. Piecewise Cubic Hermite Interpolating Polynomial (PCHIP) interpolation was employed to estimate the expected mean at intermediate concentrations (2, 3.5, 4.5, 5.5, 6, and 6.5 wt%)^24^. The corresponding standard deviation values at these concentrations were obtained by interpolating the experimental standard deviations of the neighboring points.

To mimic realistic experimental variability, Gaussian-distributed noise was added around each interpolated mean, generating 15 synthetic BDS values per new concentration. This approach allows the creation of an expanded dataset that combines experimental and synthetic data while preserving physical plausibility.

In addition to the breakdown strength, the relative permittivity of each nanocomposite formulation was measured and incorporated into the predictive framework as an auxiliary feature. Measurements were carried out using a precision LCR meter at a frequency of 50 Hz in accordance with ASTM D150. To ensure consistency, the permittivity was experimentally determined at the same filler loadings employed for the breakdown strength measurements (1, 3, 5, and 7 wt%)^21,23^.

For intermediate concentrations, the relative permittivity was estimated using effective medium mixing models, with the Maxwell–Garnett approximation adopted to generate values that preserve the physical trends of the experimental data^25^. Once both the experimental and model-derived permittivity values were obtained, they were integrated with the breakdown strength dataset and subsequently utilized as input features in the machine learning process, thereby enriching the predictive capacity of the developed framework.

The combined dataset was then used to train and evaluate five regression-based machine learning algorithms: Random Forest Regressor (RFR), Gradient Boosting Regressor (GBR), Support Vector Regressor (SVR), AdaBoost Regressor, and Extreme Gradient Boosting (XGBoost)^16,18^.

The input feature is the nanofiller concentration (wt%) and the nanofiller relative permittivity, and the target variable was the measured or augmented BDS (kV/mm). Data were randomly split into training (80%) and testing (20%) subsets, and the model performance was assessed using the coefficient of determination (R^2^), which reflects overall goodness of fit.

To gain deeper insight into the decision-making process of the trained machine learning model, the feature importance ranking, and an explainability analysis was carried out using SHAP (SHapley Additive exPlanations). These visualizations serve as a foundation for interpreting the role of concentration and permittivity in shaping the model predictions for each nanofiller system.

The best-performing model was selected and applied to a fine concentration grid of 1–7 wt% range to identify the concentration corresponding to the maximum predicted BDS which is then tested experimentally to check the model accuracy for each composite. This workflow provides a reproducible and generalizable methodology for optimizing the nanofiller loading in epoxy nanocomposites while reducing the experimental burden.

Finally, in addition to material-specific models, a cross-material machine learning model was developed to capture the relationship between the filler intrinsic permittivity, composite permittivity, concentration, and dielectric breakdown strength across all investigated systems. By replacing the categorical material type with its intrinsic permittivity, the model learns a physics-informed mapping that provides a descriptor-based framework that can be extended to additional filler systems when the same descriptor space is available, thereby offering a more universal and scalable predictive framework.

**Fig. 1:**
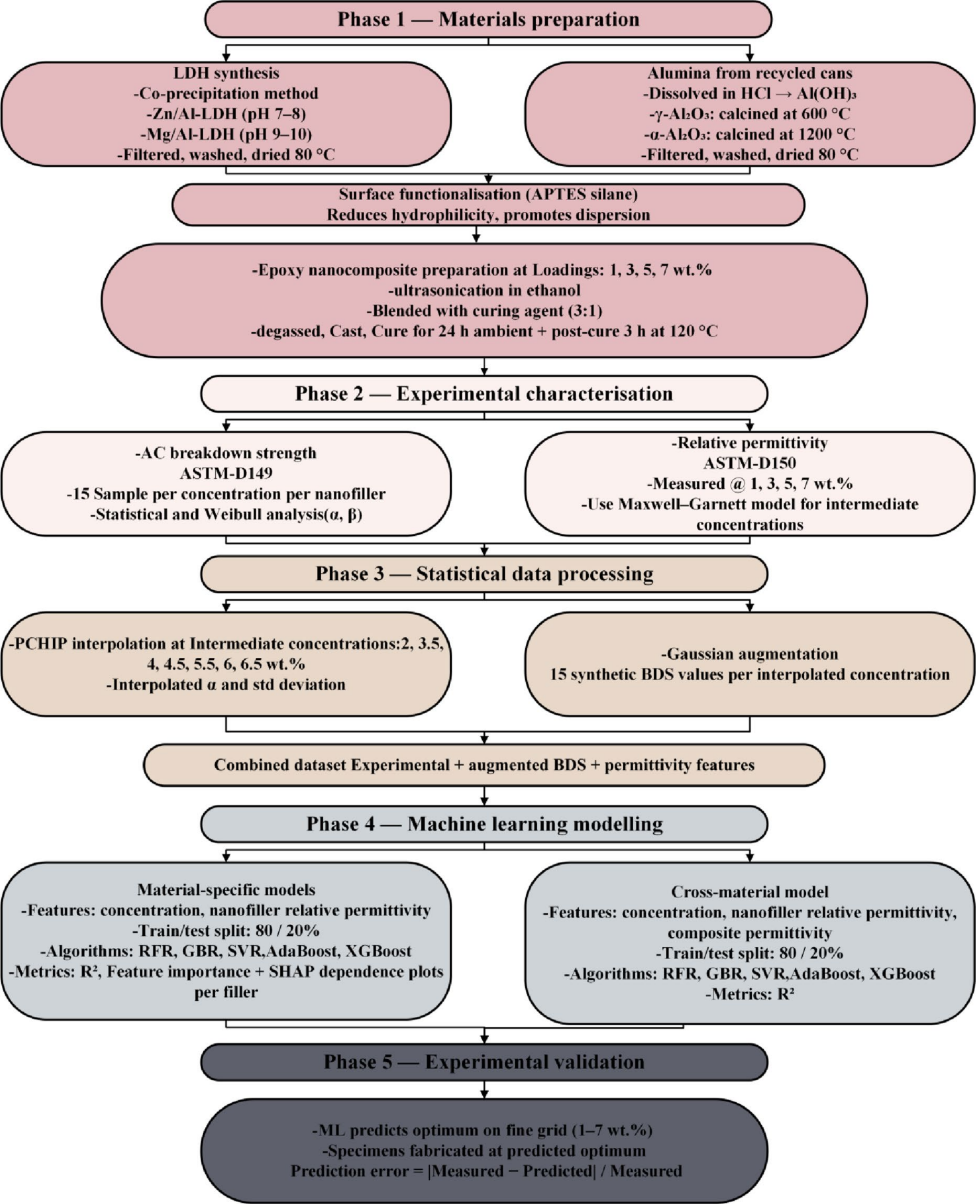
Schematic representation of the integrated experimental–statistical–machine learning framework.

In this context, the term ‘physics-informed’ refers to the deliberate incorporation of physical knowledge at the feature engineering and data generation stages of the modelling pipeline, rather than through explicit mathematical constraints embedded in the model architecture. Specifically, the Maxwell–Garnett effective medium model is employed to generate physically consistent permittivity features at intermediate concentrations, and the intrinsic filler permittivity is used as a physics-based descriptor to enable cross-material generalization. This approach is consistent with the broader use of the term in materials informatics literature, where physics-informed ML encompasses any framework in which domain knowledge actively constrains or guides the learning process. A detailed description of this methodology is presented in Fig. 1.

## Results and discussion

### Experimental results

This section presents the experimental results for the investigated epoxy nanocomposites, including the BDS, statistical reliability analysis, and relative permittivity. These results establish the physical foundation for the subsequent data processing and machine learning modelling.

The neat epoxy exhibited a baseline BDS of approximately 30 kV/mm, served as a reference for all subsequent comparisons. The incorporation of Zn/Al-LDH enhancement up to 37.4 kV/mm at 5 wt%, but a sharp decline was observed at 7 wt% (29.7 kV/mm), indicating the onset of particle agglomeration. Mg/Al-LDH exhibited a similar trend, reaching its maximum at 5 wt% (36.8 kV/mm), followed by a slight reduction at higher loading, suggesting a relatively more homogeneous dispersion compared to Zn/Al. Gamma alumina provided remarkable enhancement, peaking at 5 wt% (44.2 kV/mm), while alpha alumina exhibited the highest BDS overall, reaching 46.8 kV/mm at 5 wt% before experiencing a sharp drop at 7 wt% (36.9 kV/mm).

The results confirmed that the addition of nanofillers significantly improved the dielectric strength of the epoxy compared to that of the neat matrix. However, the improvements were strongly dependent on the type of filler and its concentration, with an optimum loading of approximately 5 wt% for all systems. These findings emphasize the necessity of identifying the optimal filler concentration rather than indiscriminately increasing the filler content, which may otherwise lead to performance deterioration due to particle agglomeration.

Given that the data augmentation process relies on the reliability of the experimental measurements, sufficient samples (15 per concentration) were used to ensure statistical robustness and reproducibility. To further validate the reliability of the obtained breakdown data, a Weibull statistical analysis was performed for each nanofiller at each loading level. This approach not only quantifies the dispersion and confidence of the breakdown measurements but also provides an accurate description of the underlying failure behavior, thereby reinforcing the credibility of the dataset used in the following augmentation and machine learning analyses.

Weibull analysis was conducted for each nanofiller at every concentration using the 15 recorded breakdown values. The two-parameter Weibull distribution provides the characteristic breakdown strength (α, scale parameter) and shape parameter (β), where a higher β indicates narrower data dispersion and greater reliability.

As shown in Table 1, the characteristic breakdown strength (α) values follow the same trends observed in the mean BDS, with the optimum performance typically achieved at intermediate loadings (around 5 wt% for Zn/Al-LDH, Mg/Al-LDH, gamma alumina, and alpha alumina). The shape parameter (β) generally remained within the range of 25–60, indicating the good reproducibility of the measurements. Higher β values, such as those observed for α-alumina at 3 wt% (β ≈ 62), reflect narrow data dispersion and strong reliability, while relatively lower β values (e.g., gamma alumina at 1 wt%) indicate broader scattering.

**Table 1:** The statistical data summary for all fillers loadings.

Nano-filler	Conc. (wt%)	Mean (kV/mm)	Std. deviation (kV/mm)	α (kV/mm)	β	*R* ^2^
Neat epoxy	0	30.08	0.91	30.51	36.83	0.94
Zn/Al-LDH	1	33.90	0.88	34.32	43.05	0.97
	3	36.06	0.90	36.49	44.47	0.93
	5	37.42	0.99	37.88	42.76	0.97
	7	29.68	0.49	29.92	65.70	0.89
Mg/Al-LDH	1	34.33	0.84	34.72	45.82	0.96
	3	35.24	0.94	35.68	42.04	0.94
	5	36.75	1.12	37.29	35.58	0.87
	7	35.12	1.21	35.69	32.15	0.93
γ-Al_2_O_3_	1	32.73	1.22	33.31	29.41	0.89
	3	38.54	1.54	39.28	26.83	0.85
	5	44.19	1.13	44.71	44.01	0.95
	7	40.09	0.73	40.72	61.29	0.95
α-Al_2_O_3_	1	34.78	1.08	35.28	36.03	0.96
	3	40.47	0.72	40.82	61.66	0.89
	5	46.79	1.67	47.60	30.37	0.87
	7	36.88	0.96	37.35	41.99	0.90

**Fig. 2:**
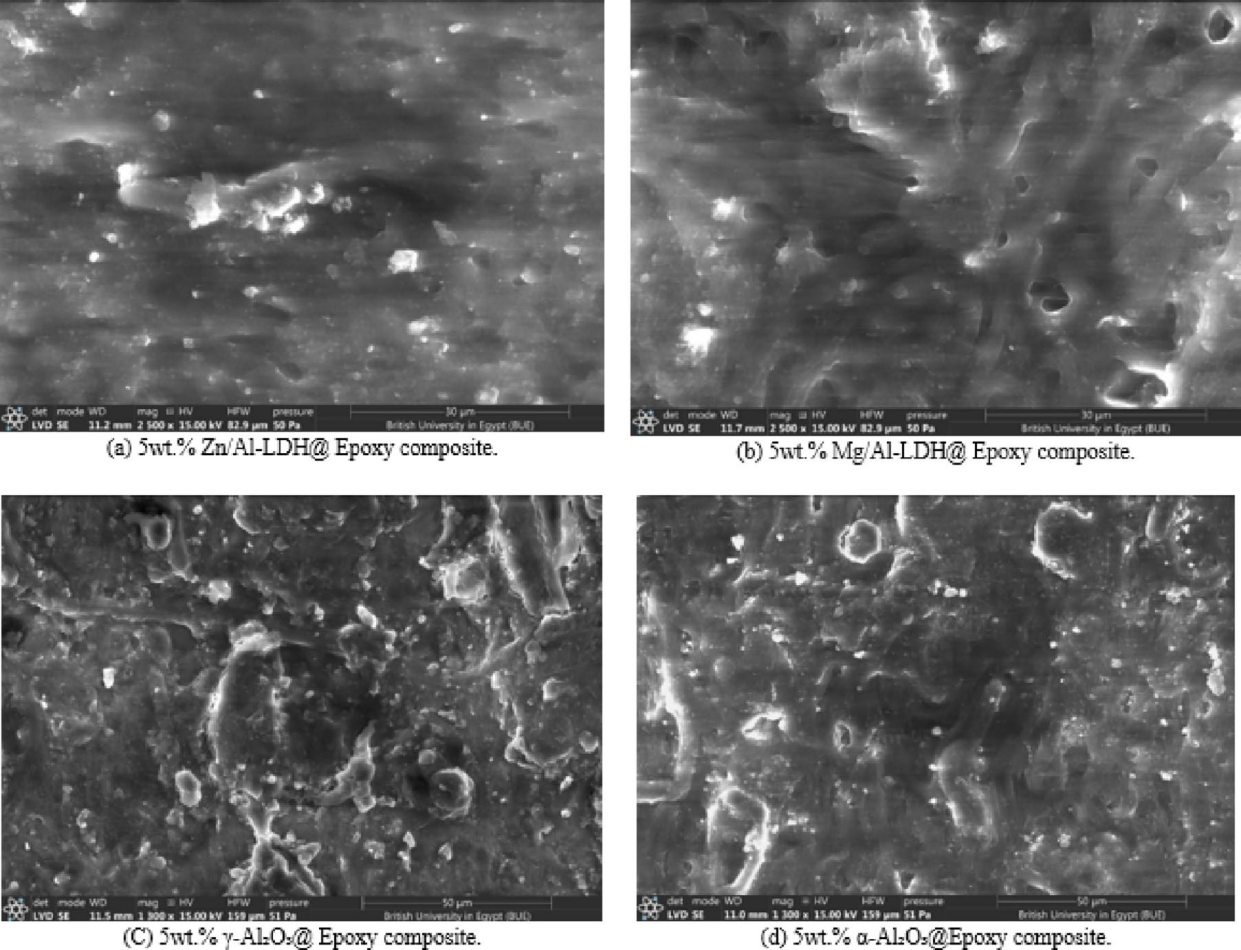
SEM images of epoxy nanocomposites at their optimal filler loading (5 wt%) for different nanofiller systems.

To further examine the microstructural characteristics of the prepared nanocomposites and verify the dispersion quality of the nanofillers, SEM analysis was conducted for all systems at their optimal filler loading (~ 5 wt%). As shown in Fig. 2, all the nanocomposite systems exhibited relatively uniform nanoparticle dispersion within the epoxy matrix, with only minor localized agglomeration. No evidence of severe or continuous agglomeration networks was observed, indicating good compatibility between the nanofillers and the epoxy matrix. This dispersion state is consistent with the observed enhancement in the breakdown strength and the high reproducibility of the experimental results, as reflected by the relatively high Weibull shape parameter (β) values and low standard deviations. Furthermore, this observation supports the optimal performance achieved at intermediate filler loadings and subsequent deterioration at higher concentrations owing to increased agglomeration effects.

The SEM observations presented in this study are intended to provide qualitative confirmation of nanoparticle dispersion and to support the physical interpretation of the dielectric breakdown behavior. Quantitative image-based descriptor extraction (e.g., particle size distribution, dispersion indices, or morphological metrics for machine learning) was intentionally beyond the scope of the present study, whose primary objective was to develop a descriptor-based framework using experimentally measured dielectric descriptors.

The observed differences in BDS enhancement across the four nanofiller systems can be further interpreted in terms of their distinct microstructural and surface characteristics. The superior performance of α-Al_2_O_3_ relative to γ-Al_2_O_3_ is attributed to its higher crystallographic density and structural order, which promote stronger interfacial bonding with the APTES-functionalized matrix, resulting in a denser interfacial region that is expected to hinder charge transport and improve dielectric reliability^13,23^. In contrast, γ-Al_2_O_3_, as a transitional alumina phase with higher surface hydroxyl density, provides a larger interfacial zone per unit volume that enhances charge trapping and charge scattering, accounting for its strong BDS enhancement despite its lower intrinsic density^23^. The comparatively moderate improvement observed in the LDH-based systems is consistent with their lamellar nanorod morphology, which generates anisotropic barrier structures that are less uniformly distributed throughout the epoxy matrix than the near-spherical alumina nanoparticles^21^. Nevertheless, the platelet geometry of LDH nanorods can introduce tortuous pathways that partially impede discharge propagation and contribute to the observed improvement over neat epoxy^11,21^. Furthermore, the common optimum concentration near 5 wt% across all investigated systems reflects a balance between increasing interfacial barrier density at moderate loadings and agglomeration-induced local field enhancement at higher concentrations. Beyond this concentration, agglomerate clusters introduce heterogeneous regions that can act as localized electrical stress concentrators, leading to progressive deterioration in breakdown performance. These mechanistic interpretations are consistent with both the experimental BDS results, and the Weibull analysis presented in Table 1, and provide a physical basis for the concentration-dependent trends subsequently captured by the machine learning models.

In addition to the breakdown strength, the relative permittivitis of the epoxy nanocomposites were experimentally measured at the same filler concentrations (1, 3, 5, and 7 wt%). This parameter was introduced as an additional physical descriptor to support the interpretation of dielectric behavior and to serve as an input feature in the subsequent machine learning analysis.

To extend the dataset beyond these discrete experimental points, mathematical mixing models were employed to describe the effective permittivity of the composite as a function of the filler and matrix permittivity. Several well-established models, including the Linear Mixing Rule, the Maxwell–Garnett approximation, and the Logarithmic model, were evaluated against the experimental results, as shown in Table 2, which describes the comparison between the predicted value of the composite affected relative permittivity and the measured values for a selected concentration. Maxwell–Garnett model described in Eq. (1) provided the closest agreement with the measured data and was therefore adopted to estimate the relative permittivity at intermediate concentrations^25^. These predicted values, together with the measured ones, were incorporated as additional input features for training the machine learning model, enabling it to learn not only from the filler concentration, but also from the dielectric response of the material.

1 $$\:{{\epsilon\:}}_{{e}{f}{f}}={{\epsilon\:}}_{{m}}\frac{{{\epsilon\:}}_{{f}}+{2{\epsilon\:}}_{{m}}+2{\varphi\:}({{\epsilon\:}}_{{f}}-{{\epsilon\:}}_{{m}})}{{{\epsilon\:}}_{{f}}+{2{\epsilon\:}}_{{m}}-{\varphi\:}({{\epsilon\:}}_{{f}}-{{\epsilon\:}}_{{m}})}$$

where ε_eff_ is the effective relative permittivity of the composite, ε_m_ is the epoxy matrix permittivity, ε_f_ is the nanofiller permittivity, and φ is the filler volume fraction.

**Table 2:** Comparison between the predicted value of the composite relative permittivity and the measured values for some of the tested concentrations.

Nano-filler	Conc. (wt%)	Measured value	Linear mixing (ε_eff_)	Maxwell–Garnett (ε_eff_)	Logarithmic model (ε_eff_)
Zn/Al-LDH	1	4.813	4.944	4.872	4.8703
Mg/Al-LDH	3	4.8540	5.37	4.9893	4.9894
γ-Al_2_O_3_	5	4.837	4.874	4.8679	4.8658
α-Al_2_O_3_	7	4.925	4.940	4.9126	4.9064

### Data processing

To overcome the limited number of tested concentrations, PCHIP interpolation was employed to generate the intermediate breakdown strength values. The shape-preserving nature of the PCHIP method is well suited to the concentration range investigated in this study. Unlike conventional cubic spline interpolation, PCHIP preserves the local monotonicity of the experimental data and avoids overshoot between adjacent data points, thereby maintaining physically realistic concentration–property trends without introducing artificial oscillations.

**Fig. 3:**
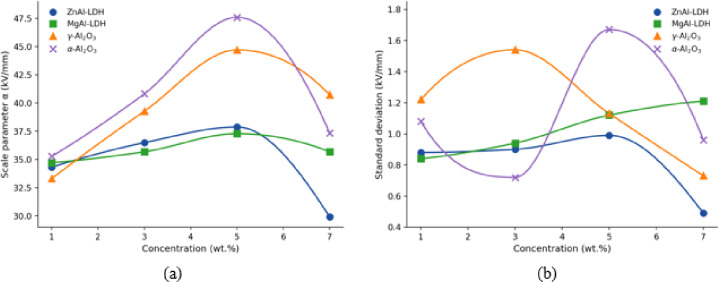
PCHIP interpolation for (**a**) scale parameter, and (**b**) standard deviation data for all nanofillers.

Interpolation was applied to both the characteristic breakdown strengths (α) obtained from the Weibull analysis and the standard deviations of the experimental data. This resulted in smooth and physically consistent curves describing the variation in BDS across the entire concentration range, as shown in Fig. 3.

The smooth interpolation assumption underlying PCHIP is physically justified for the concentration range investigated in this study (1–7 wt%). For LDH-based and alumina-based nanoparticles in epoxy matrices, percolation phenomena and abrupt phase transitions are not expected within this loading range, as percolation thresholds in such systems typically occur at substantially higher filler concentrations. This is directly supported by the experimental observations: the measured BDS values exhibit continuous unimodal trends across all four systems with no abrupt discontinuities, and the Weibull shape parameters (β = 25–65) remain within physically consistent ranges throughout, with no sudden variations indicative of microstructural regime changes.

To verify the reliability of the PCHIP-based synthetic data generation strategy, a Leave-One-Out Reconstruction (LOO-R) validation was conducted using only the experimental breakdown strength data. In each iteration, one experimentally measured concentration was temporarily removed from a given nanofiller dataset, while the remaining experimental concentration means were used to reconstruct the omitted point using PCHIP interpolation. The reconstructed value was then compared with the corresponding experimental mean BDS, and the reconstruction error was calculated.

The validation results confirmed that the interpolation-based reconstruction reproduced the internal experimental concentrations with acceptable accuracy as the reconstruction error did not exceed 7.76% for all interpolation concentrations, as shown in Table 3. The distinction between interpolation and extrapolation was explicitly considered: internal concentration points were reconstructed within the experimentally bounded domain, whereas edge concentrations required extrapolation and therefore showed larger deviations. Since the synthetic data used for ML training were generated only at intermediate concentrations within the experimentally measured range, the interpolation cases provide the most relevant assessment of augmentation reliability.

It should be emphasized that the Leave-One-Out Reconstruction analysis evaluates the internal consistency of the interpolation procedure with respect to the available experimental observations. Although the low reconstruction errors support the suitability of the PCHIP approach for generating intermediate values within the investigated concentration range, they do not independently demonstrate that the underlying concentration-property relationship is intrinsically smooth or uniquely determined. Rather, the interpolation faithfully reflects the trends supported by the available experimental measurements while remaining subject to the limitations imposed by the finite sampling density.

**Table 3:** Leave-one-out reconstruction results from experimental data.

Nanofiller	Removed Conc. (wt%)	Experimental mean BDS (kV/mm)	Reconstructed mean BDS (kV/mm)	Absolute error (kV/mm)	Reconstruction error (%)	Validation condition
Zn/Al-LDH	1.00	33.905	28.540	5.364	15.822	Extrapolation
Zn/Al-LDH	3.00	36.061	36.981	0.921	2.553	Interpolation
Zn/Al-LDH	5.00	37.421	34.958	2.463	6.581	Interpolation
Zn/Al-LDH	7.00	29.677	35.805	6.127	20.646	Extrapolation
Mg/Al-LDH	1.00	34.330	30.474	3.856	11.233	Extrapolation
Mg/Al-LDH	3.00	35.240	36.314	1.073	3.046	Interpolation
Mg/Al-LDH	5.00	36.747	35.225	1.522	4.142	Interpolation
Mg/Al-LDH	7.00	35.117	38.703	3.586	10.211	Extrapolation
γ-Al_2_O_3_	1.00	32.732	24.693	8.039	24.561	Extrapolation
γ-Al_2_O_3_	3.00	38.537	41.528	2.992	7.763	Interpolation
γ-Al_2_O_3_	5.00	44.186	41.687	2.499	5.656	Interpolation
γ-Al_2_O_3_	7.00	40.089	49.678	9.590	23.921	Extrapolation
α-Al_2_O_3_	1.00	34.775	28.341	6.434	18.503	Extrapolation
α-Al_2_O_3_	3.00	40.475	43.138	2.664	6.581	Interpolation
α-Al_2_O_3_	5.00	46.791	43.726	3.066	6.552	Interpolation
α-Al_2_O_3_	7.00	36.883	43.694	6.811	18.467	Extrapolation

After validation of the PCHIP method, the characteristic breakdown strength (α) was estimated at intermediate concentrations that were not experimentally tested from fitting curves. It should be noted that the values presented in Table 4 are not intended as exhaustive datasets for all nanofillers, but rather as representative snapshots derived from the PCHIP fitting process. For each material, interpolated α and standard deviation values were extracted at predefined intermediate concentrations (2, 3.5, 4.5, 5.5, 6, and 6.5 wt%). Similarly, the corresponding standard deviation values were interpolated, ensuring that both the mean trend and the expected variability of the data were consistently represented across the entire loading range.

The variation observed in the standard deviation values in Fig. 3b does not follow a single monotonic trend across all nanofillers, and this behavior reflects the combined influence of the stochastic nature of dielectric breakdown and the material-dependent dispersion characteristics of each nanocomposite system.

In general, dielectric breakdown is inherently probabilistic, and the measured variability strongly depends on the local electric field distribution and microstructural uniformity. However, the evolution of the standard deviation with filler concentration differs among nanofillers due to variations in the particle morphology, interfacial interaction, and dispersion stability. For instance, in some systems, such as LDH-based nanocomposites, the variation is relatively smooth, indicating stable dispersion and gradual changes in the microstructure. In contrast, alumina-based systems exhibit more pronounced fluctuations, particularly near intermediate concentrations, where competing effects such as improved interfacial interaction and the onset of localized clustering coexist. This leads to non-monotonic behavior in the standard deviation.

At higher loadings, agglomeration effects may dominate in certain systems, increasing variability, whereas in others, partial restructuring or redistribution of particles can locally reduce dispersion-induced fluctuations, explaining the observed differences in trends. Therefore, the variation in the standard deviation should be interpreted as a material-specific response rather than a universal trend, governed by the interplay between dispersion quality, particle interaction, and electric field heterogeneity.

These interpolated values form the basis for the next step, where 15 synthetic data points are generated around each predicted α using Gaussian-distributed noise. This approach is justified by the physical nature of dielectric breakdown, which typically follows a Gaussian-like distribution, with the interpolated standard deviation serving as the constraint for the spread of the generated values.

The resulting processed dataset, combining both experimental and augmented data, provides a continuous and statistically consistent representation of the concentration–BDS relationship, enabling more reliable machine learning model training.

It is important to note that the augmented data were used solely to enhance model training and predictive capability, whereas all physical interpretations were based exclusively on experimental measurements.

**Table 4:** Snapshots for the predicted scale parameter and Std derived from the PCHIP fitting process.

Nanofiller	Conc. (wt%)	Predicted_α (kV/mm)	Predicted Std. deviation (kV/mm)
Zn/Al-LDH	3.5	36.95	0.92
	5.5	37.23	0.95
	6.5	32.94	0.68
Mg/Al-LDH	2.0	35.13	0.88
	4.5	37.09	1.08
	6.0	36.39	1.17
γ-Al_2_O_3_	2.0	36.36	1.47
	4.5	44.13	1.25
	6.0	43.80	0.93
α-Al_2_O_3_	3.5	42.74	0.87
	4.0	44.97	1.19
	5.5	46.88	1.52

### Model development

To develop a reliable predictive framework for dielectric breakdown strength (BDS), a comparative analysis was conducted using five machine learning regression models: Random Forest Regressor (RFR), Gradient Boosting Regressor (GBR), AdaBoost Regressor, Extreme Gradient Boosting (XGBoost), and Support Vector Regressor (SVR). These models were trained using the processed dataset described in the previous section, which included both the experimental and augmented data.

**Fig. 4:**
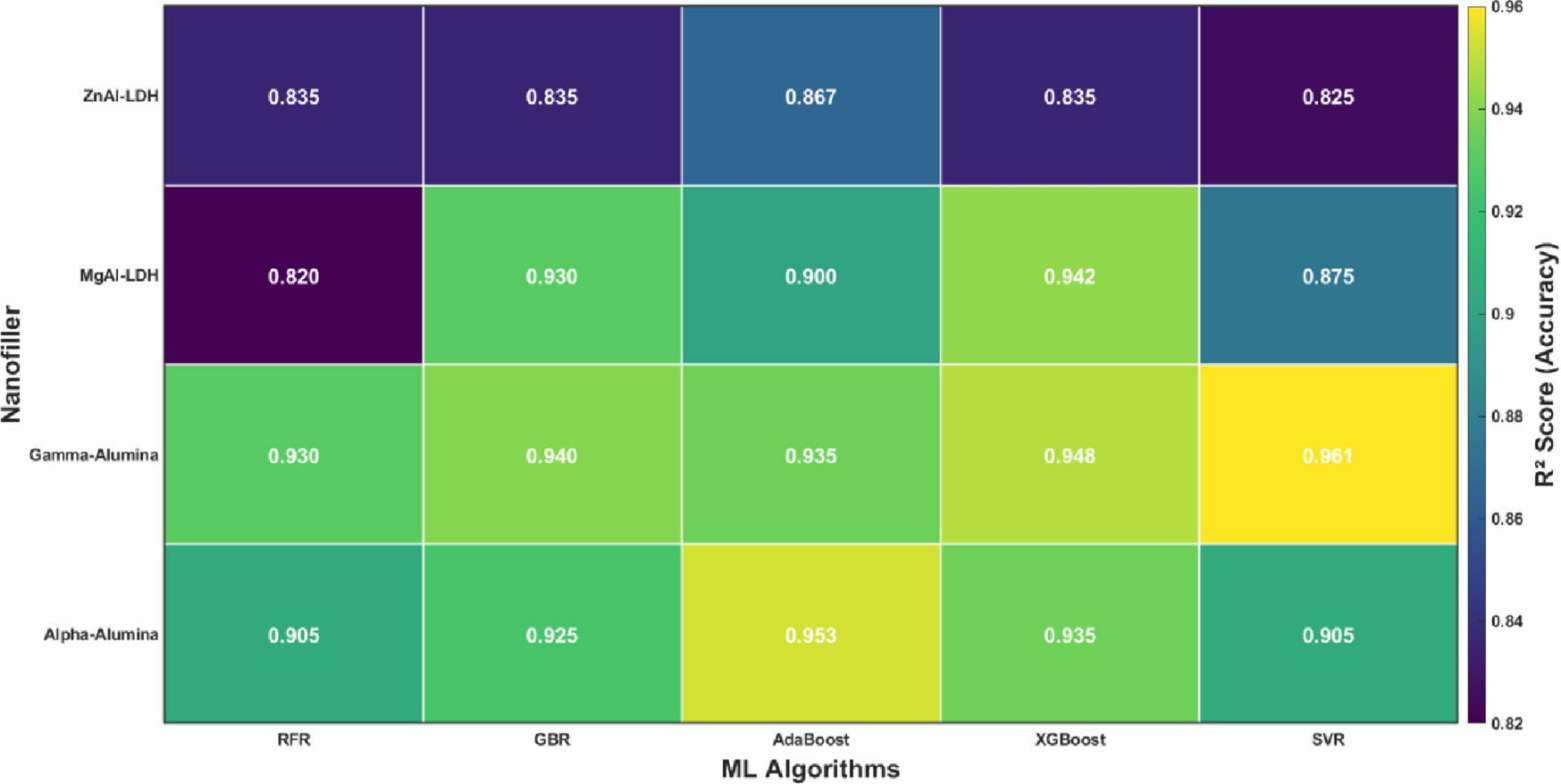
Heatmap comparing the predictive accuracy (R^2^) of ML models across different nanofiller systems.

Figure 4 presents a heatmap comparing the predictive accuracy (R^2^) of the ML models across different nanofiller systems. Each system represents the complete dataset, including all investigated filler loadings (1–7 wt%), incorporating both experimental and augmented data. The coefficient of determination (R^2^) was used as the primary metric to evaluate the model accuracy.

To provide a more comprehensive assessment beyond the coefficient of determination, Table 5 summarizes the mean absolute error (MAE) and root mean square error (RMSE) for all models across the four nanofiller systems. Overall, the error metrics corroborated the trends observed in the R^2^ comparison; the best-performing model for each system consistently achieved the lowest prediction errors. Specifically, XGBoost attained the minimum MAE and RMSE for Mg/Al-LDH (0.652 and 0.774 kV/mm, respectively), AdaBoost yielded the lowest errors for both Zn/Al-LDH (MAE = 0.671, RMSE = 0.872 kV/mm) and α-Al_2_O_3_ (MAE = 0.921, RMSE = 0.978 kV/mm), and SVR demonstrated superior error minimization for γ-Al_2_O_3_ (MAE = 0.862, RMSE = 1.020 kV/mm), consistent with its highest R^2^ among all evaluated configurations. Notably, the MAE and RMSE values across all models remained within a narrow and physically acceptable range, confirming stable generalization without significant overfitting.

**Table 5:** Performance metrics of machine learning models for dielectric strength prediction.

Material	Model	*R* ^2^	MAE (kV/mm)	RMSE (kV/mm)
Zn/Al-LDH	RFR	0.834	0.757	0.975
	GBR	0.834	0.757	0.974
	AdaBoost	0.867	0.671	0.872
	XGBoost	0.834	0.757	0.974
	SVR	0.827	0.769	0.994
Mg/Al-LDH	RFR	0.814	0.870	0.875
	GBR	0.921	0.867	0.864
	AdaBoost	0.897	0.712	0.872
	XGBoost	0.942	0.652	0.774
	SVR	0.867	0.740	0.794
γ-Al_2_O_3_	RFR	0.928	0.871	1.050
	GBR	0.938	0.869	1.049
	AdaBoost	0.931	0.891	1.101
	XGBoost	0.942	0.862	1.010
	SVR	0.959	0.862	1.020
α-Al_2_O_3_	RFR	0.901	1.083	1.327
	GBR	0.921	1.082	1.328
	AdaBoost	0.949	0.921	0.978
	XGBoost	0.932	1.082	1.328
	SVR	0.901	1.103	1.330

It is important to note that the R^2^ values reported here reflect model performance on a test set drawn from the same combined (experimental + augmented) distribution used for training. Since the augmented data points are mathematically derived from the experimental measurements via PCHIP interpolation, a degree of correlation exists between training and testing subsets. Accordingly, the reported R^2^ values should be interpreted as indicators of the model’s interpolation accuracy within the experimentally bounded concentration range, rather than as estimates of fully independent predictive performance. The most rigorous validation conducted on physically fabricated specimens at concentrations never seen by the model is presented in “Model validation”.

The relationship between concentration and BDS in epoxy nanocomposites is inherently nonlinear and non-monotonic. Across all investigated systems, dielectric breakdown strength increases with nanofiller loading up to an optimum concentration and subsequently decreases at higher loadings due to agglomeration-related effects. This concentration-dependent inflection behavior represents a complex response surface that is challenging to capture using simpler regression approaches.

Tree-based ensemble methods, including XGBoost and Gradient Boosting Regression, are particularly well suited to this type of problem because their sequential learning strategy allows them to progressively focus on difficult-to-model regions of the descriptor space. In addition, tree-based learners naturally partition the concentration space into multiple regimes and can effectively capture local interactions, threshold-like transitions, and concentration-dependent performance inflections without requiring predefined functional relationships. Furthermore, the regularization mechanisms incorporated within XGBoost provide an additional advantage when working with relatively small datasets by improving generalization and reducing the risk of overfitting. Consequently, the superior performance of the best-performing models across the investigated systems can be interpreted as a reflection of their ability to capture the nonlinear concentration–property relationships and concentration-dependent performance transitions governing dielectric breakdown behavior^26–28^.

The predictive performances of the five applied models on the Zn/Al-LDH dataset were illustrated in Fig. 4. AdaBoost exhibited the best accuracy (R^2^ = 0.867); therefore, it was selected for the interpretability analysis.

**Fig. 5:**
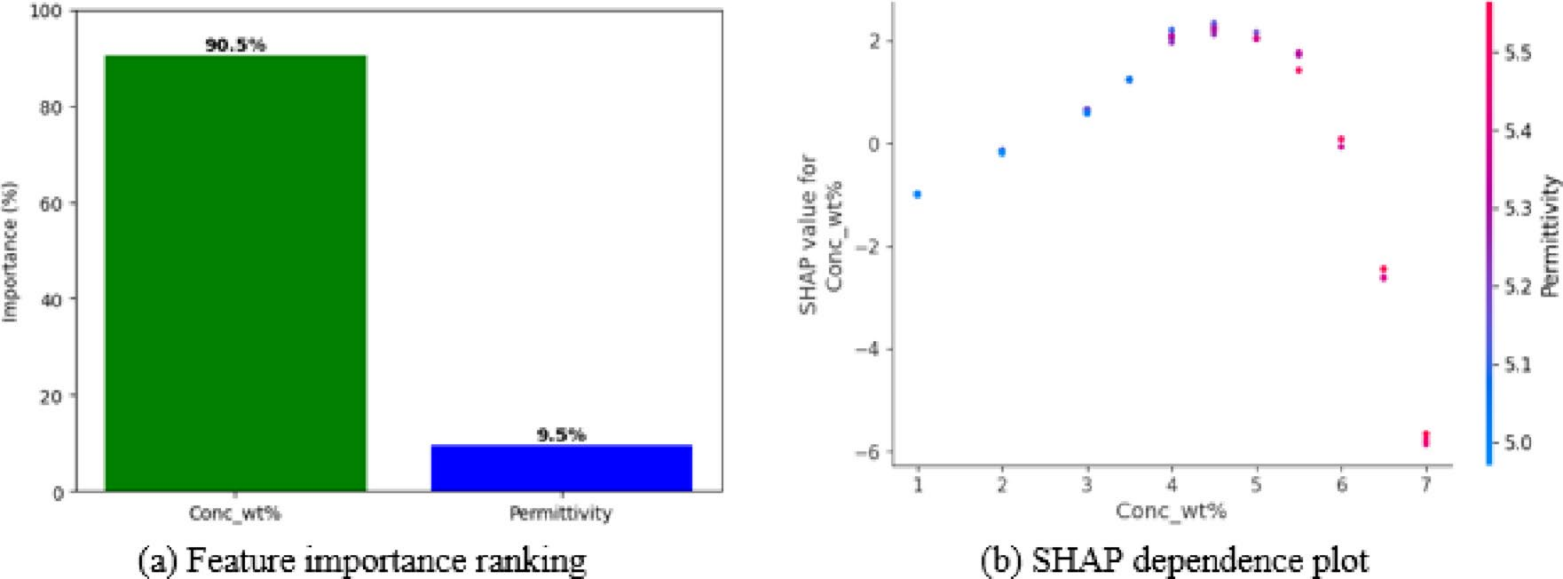
Feature importance and SHAP interpretability analysis for Zn/Al-LDH model predictions.

The feature importance ranking (Fig. 5a) indicates that concentration is the most influential predictor (> 90%), whereas permittivity contributes only marginally. The SHAP dependence plot (Fig. 5b) provides further insight, showing a non-linear trend where moderate filler loadings (≈ 3–5 wt%) increase the predicted BDS, whereas excessive concentrations (≥ 6 wt%) strongly reduce it, consistent with the agglomeration effect observed experimentally. This demonstrates that, although permittivity plays a supportive role, concentration remains a critical factor governing the BDS performance.

**Fig. 6:**
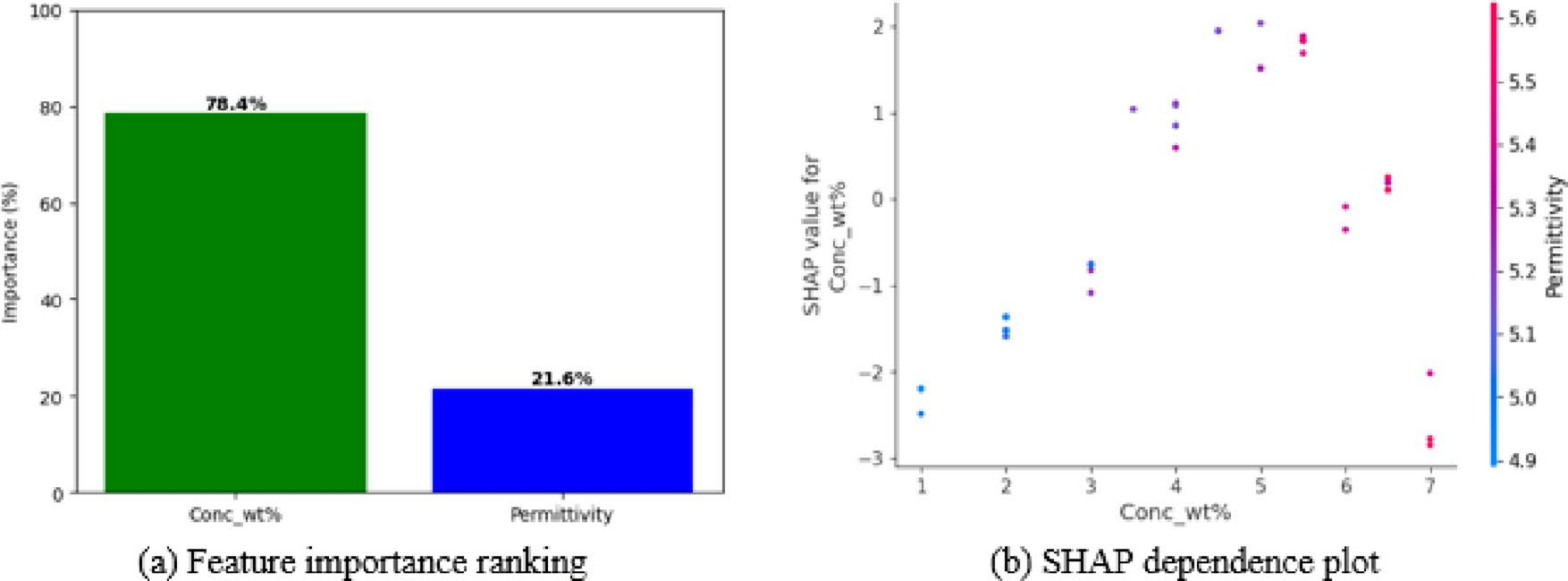
Feature importance and SHAP interpretability analysis for Mg/Al-LDH model predictions.

Unlike the Zn/Al system, the Mg/Al-LDH composites exhibited a more intricate predictive behavior. As shown in Fig. 4, XGBoost provided the most accurate forecasts (R^2^ = 0.942), while other ensemble models yielded slightly weaker but still acceptable results. The feature analysis in (Fig. 6a) indicated that the concentration remains the key driver (~ 78%), yet permittivity plays a noticeably larger role (~ 22%), suggesting that dielectric properties carry additional weight in this system. The SHAP dependence plot (Fig. 6b) pointed to a non-linear relationship, where moderate filler contents (≈ 4–5 wt%) enhanced BDS, while higher fractions reduced it due to agglomeration.

**Fig. 7:**
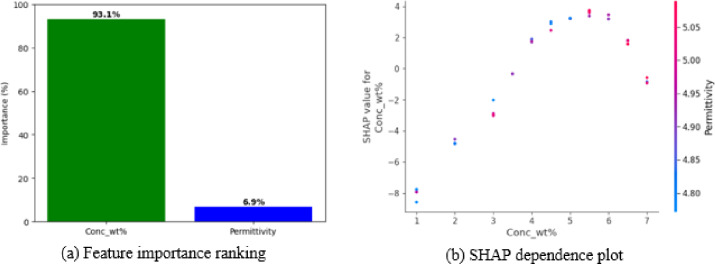
Feature importance and SHAP interpretability analysis for γ-Al_2_O_3_ model predictions.

The analysis of the γ-Al_2_O_3_ composites revealed a striking imbalance in the feature contributions. As illustrated in (Fig. 7a), the concentration overwhelmingly governs the model predictions (~ 93%), while permittivity adds only a marginal effect (~ 7%). This dominance is reflected in the SHAP dependence plot (Fig. 7b), where the BDS increases steadily with filler content up to about 5.5 wt% before declining at higher levels, consistent with the onset of agglomeration. Despite this skewed feature distribution, the models still delivered high accuracy (Fig. 4), particularly SVR and XGBoost, indicating that BDS in γ-Al_2_O_3_ systems is predominantly concentration-driven with limited influence from the dielectric permittivity.

**Fig. 8:**
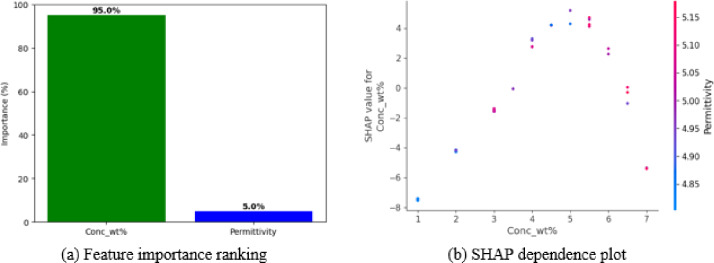
Feature importance and SHAP interpretability analysis for α-Al_2_O_3_ model predictions.

Finally, for α-Al_2_O_3_ composites, AdaBoost emerged as the most effective model with R^2^ = 0.949 and the lowest error metrics, while other ensembles, such as XGBoost and GBR also performed reliably (Fig. 4). The interpretability analysis in Fig. 8 confirms that the concentration is overwhelmingly the decisive variable (~ 95%), whereas permittivity plays only a negligible role (~ 5%). The SHAP dependence plot illustrates a non-linear effect, where the BDS improves up to around 5 wt% loading before deteriorating at higher contents, a trend that mirrors agglomeration phenomena. Thus, in α-Al_2_O_3_ systems, breakdown strength is primarily dictated by filler loading, with permittivity offering little additional explanatory power.

The concentration-dependent trend identified by the SHAP dependence analysis can be further interpreted in terms of the underlying interfacial micromechanics governing dielectric breakdown behavior. At moderate nanofiller concentrations, the increasing interfacial area between the nanoparticles and the epoxy matrix promotes charge trapping, suppresses charge transport, and enhances resistance to electrical breakdown. Consequently, the positive SHAP values observed near the optimum concentration reflect the beneficial contribution of well-dispersed nanoparticles and the associated interfacial barrier network.

However, as the nanofiller concentration increases beyond approximately 5 wt%, the probability of particle agglomeration increases significantly, leading to the formation of localized clusters and increased microstructural heterogeneity. Owing to the permittivity property mismatch between these agglomerates and the surrounding epoxy matrix, localized electric-field intensification may develop at the cluster-matrix interfaces. Furthermore, imperfect interfacial bonding around agglomerated regions can promote the formation of voids and electrically weak zones with reduced local dielectric strength.

Under high-voltage stress, these regions become favorable sites for space-charge accumulation, partial discharge initiation, and the subsequent development of microscopic electrical trees. As a result, the detrimental effects of agglomeration-induced field distortion and defect formation progressively outweigh the beneficial charge-trapping and barrier effects associated with well-dispersed nanoparticles. This transition provides a physical explanation for the reduction in breakdown strength captured by the SHAP dependence plots at concentrations above the optimum loading.

It is also worth noting that the relatively low SHAP importance assigned to composite permittivity across all investigated systems (5–22%) suggests that the model predictions are primarily governed by nanofiller concentration rather than by the Maxwell–Garnett-derived permittivity feature. Consequently, although uncertainties associated with the Maxwell–Garnett approximation may influence the estimated permittivity values, the SHAP analysis provides a practical indication that these uncertainties are unlikely to dominate the overall model predictions. This interpretation should not be regarded as a formal uncertainty propagation analysis but rather as supporting evidence consistent with the limited contribution of permittivity within the trained models.

### Model validation

Building on the model performance evaluation presented in the previous section, the best-performing model for each nanofiller system was employed to predict the optimal filler concentration that maximized the dielectric breakdown strength (BDS). This step shifts the focus from model comparison to practical applications, enabling direct identification of the optimum operating conditions for each nanocomposite.

**Fig. 9:**
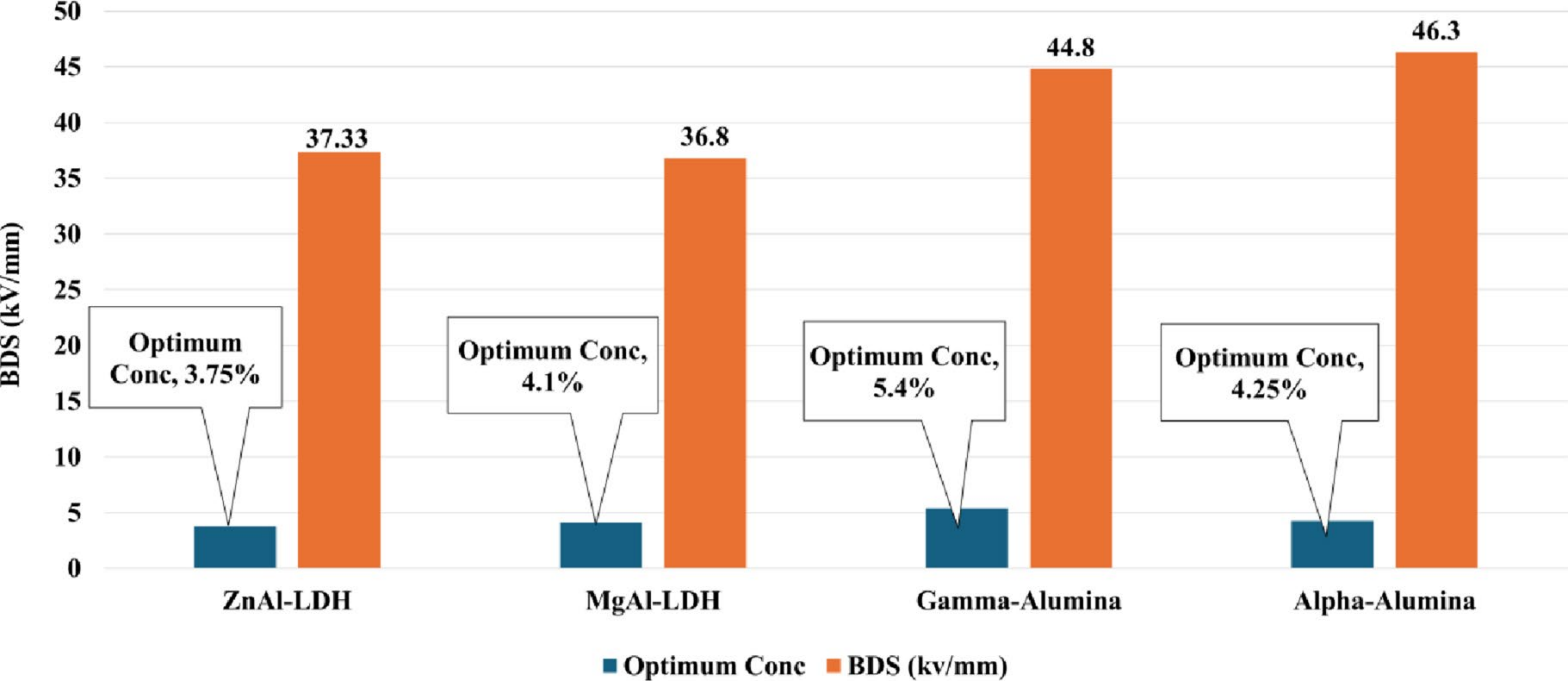
The predicted optimum concentration and the corresponding BDS for each nanofiller according to the best performed ML model.

The predicted optimal concentrations and their corresponding BDS values are summarized in Fig. 9. The Zn/Al-LDH and Mg/Al-LDH nanocomposites exhibited their maximum performance at relatively low loadings (approximately 3.75–4.1 wt%), which is consistent with the tendency of layered double hydroxides to agglomerate at higher concentrations. In contrast, the γ-Al_2_O_3_ and α-Al_2_O_3_ systems reached their optimum at moderate loadings (approximately 5.4 wt% and 4.25 wt%, respectively), with α-Al_2_O_3_ achieving the highest breakdown strength (~ 46.3 kV/mm). These results confirm that the developed models successfully captured the filler-dependent behavior and the existence of an optimal concentration for each system.

It is important to note that all predicted optimum concentrations lie within the experimentally investigated concentration range (1–7 wt%). Consequently, the reported optimization results correspond to interpolation within the experimentally bounded domain rather than extrapolation beyond the available data. This distinction increases the reliability of the predicted optimum concentrations and ensures consistency with the validated interpolation framework adopted in this study.

To validate the reliability of the machine learning predictions, specimens were fabricated at the concentrations predicted to yield the maximum breakdown strength for each nanofiller system. Breakdown tests were then conducted under the same ASTM-D149 procedure used in the experimental campaign. Finally, the predicted values were finally compared with the measured breakdown strengths according to Eq. (2):

2 $$\mathrm{Error}\,(\%)=\frac{\left|\mathrm{Measured\ value}-\mathrm{Predicted\ value}\right|}{\mathrm{Measured\ value}}\times 100$$

**Fig. 10:**
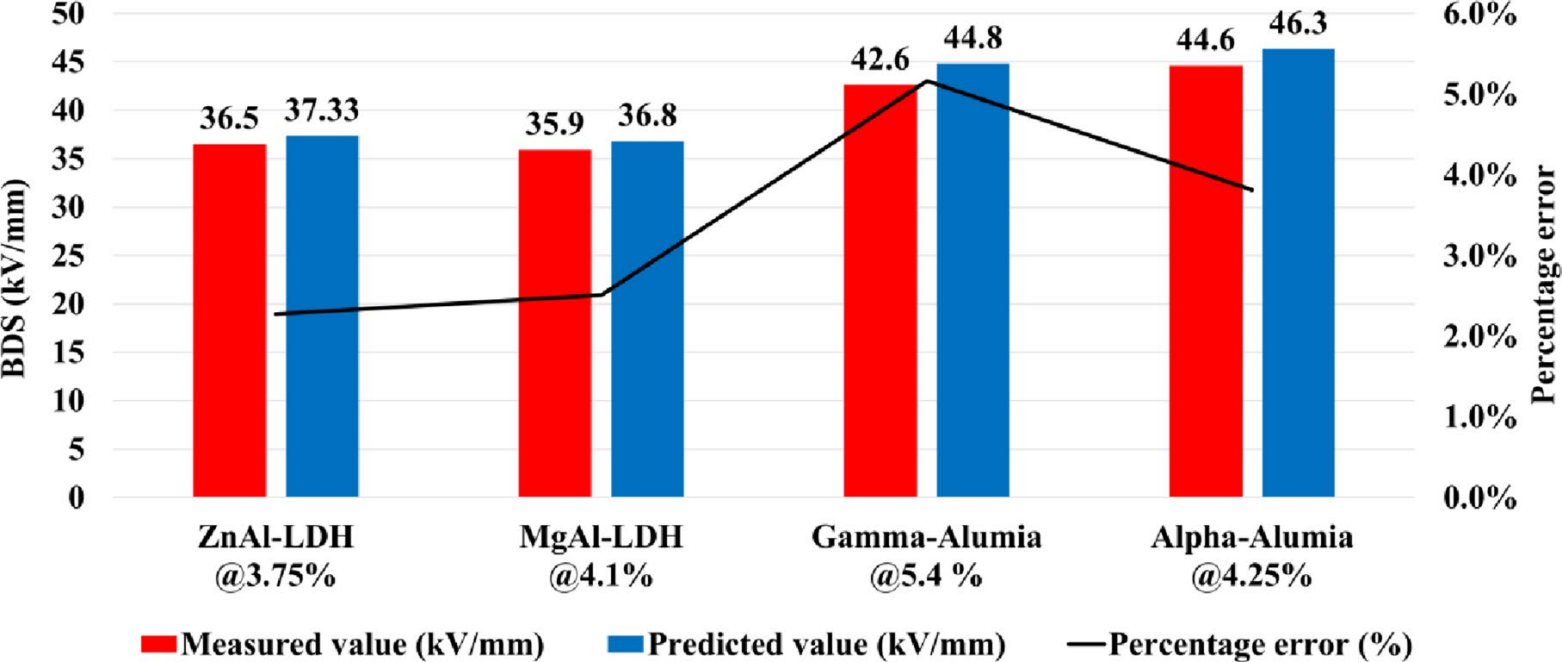
Measured and predicted breakdown strength values at the optimum concentrations for all nanocomposites, with the corresponding prediction error (%).

A comparison between the predicted and experimentally measured values is presented in Fig. 10. The results showed excellent agreement between the model predictions and experimental measurements, with prediction errors consistently below 5.2%.

This close agreement demonstrates that the proposed framework, combining statistically robust experimental data, physically constrained data augmentation, and machine learning modeling, can accurately predict the optimal filler concentration. Moreover, successful experimental validation confirms that the model captures the underlying physical behavior of dielectric breakdown rather than merely fitting the dataset.

### Cross-material machine learning model

To extend the predictive capability beyond individual nanofiller systems, a cross-material machine learning model was developed to capture the overall relationship between the key physical parameters and the BDS of epoxy-based nanocomposites. Unlike the material-specific models presented earlier, which were trained separately for each nanofiller using filler concentration and composite relative permittivity as input features, the cross-material model integrated the entire dataset of all investigated fillers into a unified framework. In this model, three input features were used: the nanofiller intrinsic permittivity, filler concentration, and composite relative permittivity. By replacing the categorical material identity with its intrinsic permittivity, the model establishes a physics-informed, material-independent mapping between input features and breakdown strength.

This formulation provides a descriptor-based framework that captures transferable relationships across the investigated nanofiller systems and can be extended to additional fillers described within the same physically meaningful descriptor space.

Among the evaluated algorithms, SVR achieved the highest accuracy (R^2^ = 0.919), whereas AdaBoost showed the weakest performance.

Although the R^2^ values of the cross-material model were slightly lower than those obtained from the material-specific models, they remained within a high accuracy range (0.872–0.919). This slight reduction was expected, as the cross-material model was trained on a more diverse and heterogeneous dataset. Despite this, the model successfully captured the overall trends across different nanofillers and maintained strong predictive performance.

**Fig. 11:**
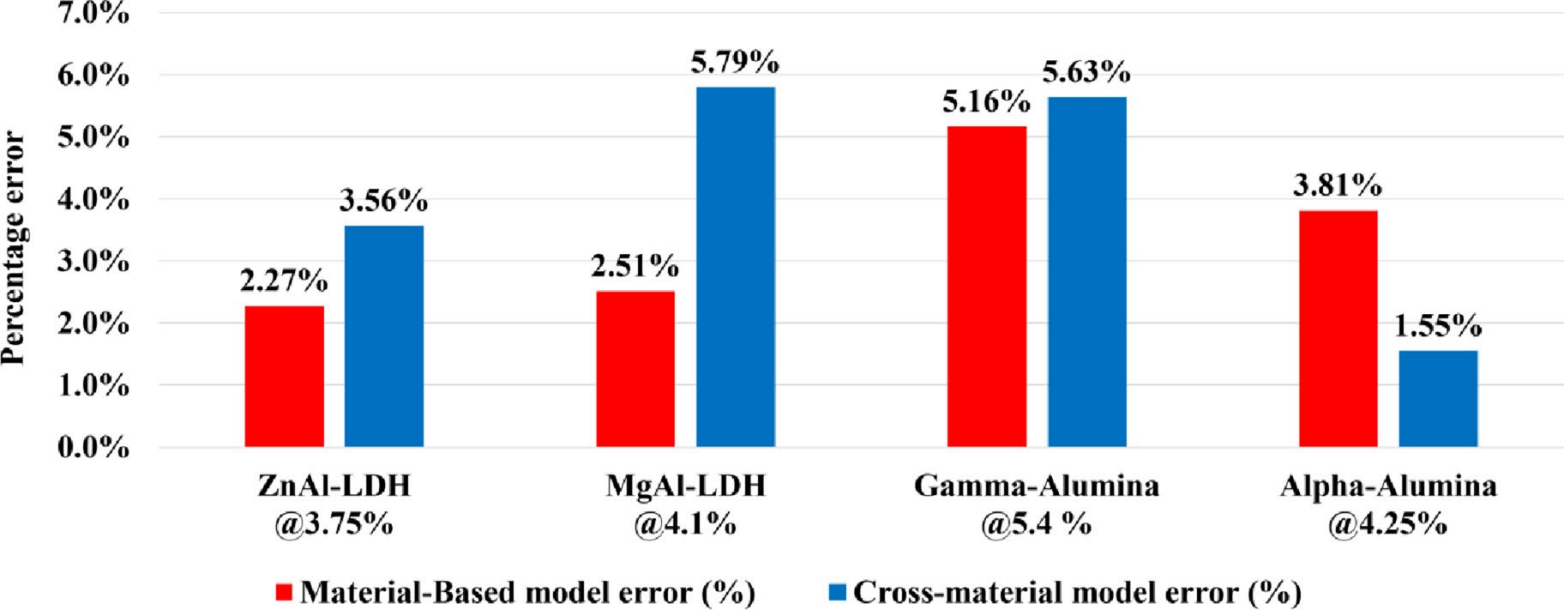
Comparison of prediction errors between the material-based models and the cross-material model for different nanofiller systems at their optimal concentrations.

A direct comparison between the experimental measurements, material-specific models, and cross-material model is presented in Table 6; Fig. 11. The model produced predictions that closely matched the experimental results, with prediction errors ranging from 1.55% to 5.79%. Although the material-specific models achieved slightly lower errors in some cases, the cross-material model provided consistently accurate predictions across all systems using a single unified model.

These results confirm that this model effectively captures the underlying relationships between the dielectric properties and breakdown strength across multiple material systems. Furthermore, its ability to deliver accurate predictions using a unified framework highlights its robustness, generalization capability, and potential practical applicability in predicting the optimal performance of new or unexplored nanofiller systems.

**Table 6:** Comparison between experimental, material-based models, and cross-material model predictions for different nanofiller systems at their optimal concentrations.

Sample	Measured value (kV/mm)	Material-based model		Cross-material model	
		Predicted value (kV/mm)	Prediction error (%)	Predicted value (kV/mm)	Prediction error (%)
Zn/Al-LDH @ 3.75%	36.5	37.33	2.27%	37.8	3.56%
Mg/Al-LDH @ 4.1%	35.9	36.8	2.51%	37.98	5.79%
γ-Al_2_O_3_ @ 5.4%	42.6	44.8	5.16%	45	5.63%
α-Al_2_O_3_ @ 4.25%	44.6	46.3	3.81%	45.29	1.55%

## Limitations of the present framework

Despite the promising predictive performance achieved by the proposed machine learning framework, several limitations should be acknowledged.

First, the framework was intentionally designed around a limited set of physically meaningful and readily accessible dielectric descriptors, including nanofiller concentration and permittivity-related parameters. While this design choice substantially reduces experimental requirements and facilitates practical implementation, other potentially influential microstructural descriptors, such as particle size, specific surface area, aspect ratio, dispersion characteristics, and interfacial morphology, were not explicitly incorporated into the current model. The integration of such descriptors may further improve predictive capability and provide a more comprehensive representation of structure-property relationships.

Second, the interpolation-based augmentation strategy adopted in this work assumes smooth evolution of dielectric breakdown strength between experimentally measured concentrations. Although this assumption was supported by the observed experimental trends and the Leave-One-Out Reconstruction validation results, caution should be exercised when applying the methodology to systems exhibiting abrupt percolation behavior, discontinuous phase transitions, or severe concentration-dependent microstructural changes.

In addition, the Maxwell–Garnett approximation used for intermediate permittivity estimation is associated with assumptions of dilute and well-dispersed inclusions. While good agreement with experimental measurements was observed throughout the investigated concentration range, including higher filler loadings, alternative effective-medium models may be required for systems exhibiting stronger particle–particle interactions or significantly higher nanofiller concentrations.

Finally, although the proposed Cross-Material Model was developed using physically meaningful descriptor-based inputs rather than categorical material identity, its applicability beyond the nanofiller systems investigated in this study has not yet been validated using a completely independent nanofiller family. It should be emphasized that the primary contribution of this work is the development of a transferable optimization framework rather than a universal predictive model. The descriptor-based nature of the framework provides a rational basis for potential extension to additional nanofiller systems; however, rigorous verification of such transferability requires future validation on unseen nanofiller families and may benefit from the incorporation of additional microstructural descriptors, such as particle size, specific surface area, and dispersion characteristics.

Despite these limitations, the proposed framework demonstrates that accurate prediction of optimal nanofiller concentration and dielectric breakdown strength can be achieved using a relatively small experimental dataset and a limited set of physically meaningful dielectric descriptors. The methodology therefore provides a practical and transferable low-data optimization workflow that can serve as a foundation for future descriptor-enriched and externally validated machine learning models for dielectric nanocomposite design.

## Conclusion

This study proposed an integrated experimental–statistical–machine learning framework for optimizing the dielectric breakdown strength (BDS) of epoxy-based nanocomposites for high-voltage insulation applications. Four nanofiller systems—Zn/Al-LDH, Mg/Al-LDH, γ-Al_2_O_3_, and α-Al_2_O_3_—were systematically investigated, including recycled alumina nanoparticles derived from aluminum beverage cans. The main findings are summarized as follows:

1. Nanofiller incorporation significantly enhanced the dielectric strength of epoxy, with the optimum performance generally achieved near 5 wt% loading. The highest BDS values were obtained for α-Al_2_O_3_ (46.8 kV/mm) and γ-Al_2_O_3_ (44.2 kV/mm), corresponding to improvements of up to approximately 56% compared with neat epoxy. LDH-based systems also demonstrated stable enhancements of approximately 25%, while excessive loading caused performance deterioration because of agglomeration effects.
2. The PCHIP-based data augmentation strategy successfully expanded the dataset by generating physically consistent intermediate concentrations. Leave-One-Out Reconstruction validation confirmed the reliability of the interpolation approach, with reconstruction errors below 7.76% for all internal interpolation points.
3. The developed material-specific machine learning models achieved strong predictive performance, with R^2^ values ranging from 0.867 to 0.961 and low MAE/RMSE values across all investigated systems.
4. Feature importance and SHAP analyses revealed that filler concentration was the dominant factor controlling BDS predictions, whereas composite permittivity provided a secondary but physically meaningful contribution, particularly for Mg/Al-LDH nanocomposites.
5. Experimental validation performed at the ML-predicted optimum concentrations showed prediction errors below 5.2%, confirming that the developed framework accurately captures the underlying dielectric breakdown behavior rather than merely fitting the dataset.
6. A cross-material physics-informed model was successfully developed by replacing categorical material identity with intrinsic filler permittivity as a physically meaningful descriptor. The cross-material model achieved R^2^ = 0.919 with prediction errors below 6% across all investigated nanofillers, demonstrating strong scalability and cross-material prediction capability.

Collectively, the proposed framework provides a robust and scalable data-driven methodology for optimizing epoxy nanocomposite insulation systems while significantly reducing experimental effort. The developed approach offers strong potential for the design and optimization of advanced GIS/GIL spacer materials requiring high dielectric reliability and accurate breakdown strength prediction.

## Data Availability

Data will be shared on a reasonable request.

## Code availability

The python codes used for machine learning model training in this study have been deposited in the Zenodo repository and are publicly available at: 10.5281/zenodo.20630671.
